# A binary prototype for time-series surveillance and intervention

**DOI:** 10.1016/j.epidem.2025.100866

**Published:** 2025-11-11

**Authors:** Jason Olejarz, Till Hoffmann, Alex Zapf, Douaa Mugahid, Ross Molinaro, Chadwick Brown, Artem Boltyenkov, Taras Dudykevych, Ankit Gupta, Marc Lipsitch, Rifat Atun, Jukka-Pekka Onnela, Sarah Fortune, Rangarajan Sampath, Yonatan H. Grad

**Affiliations:** a Department of Immunology and Infectious Diseases, Harvard T. H. Chan School of Public Health, Boston, MA 02115, USA; b Department of Biostatistics, Harvard T. H. Chan School of Public Health, Boston, MA 02115, USA; c Department of Epidemiology, Harvard T. H. Chan School of Public Health, Boston, MA 02115, USA; d Siemens Healthcare Diagnostics, Inc., Tarrytown, NY 10591, USA; e Department of Global Health Systems, Department of Health Policy and Management, Harvard T. H. Chan School of Public Health, Boston, MA 02115, USA

**Keywords:** Optimization, Cost–benefit analysis, Anomaly detection, Quality control, Healthcare, Infectious diseases

## Abstract

Despite much research on early detection of anomalies from surveillance data, a systematic framework for appropriately acting on these signals is lacking. We addressed this gap by formulating a hidden Markov-style model for time-series surveillance, where the system state, the observed data, and the decision rule are all binary. We incur a delayed cost, *c*, whenever the system is abnormal and no action is taken, or an immediate cost, *k*, with action, where *k < c*. If action costs are too high, then surveillance is detrimental, and intervention should never occur. If action costs are sufficiently low, then surveillance is detrimental, and intervention should always occur. Only when action costs are intermediate and surveillance costs are sufficiently low is surveillance beneficial. Our equations provide a framework for assessing which approach may apply under a range of scenarios and, if surveillance is warranted, facilitate methodical classification of intervention strategies. Our model thus offers a conceptual basis for designing real-world public health surveillance systems.

## 1. Background

Temporal surveillance is integral to medicine and public health. A classic example is the prediction of expected numbers of cases of an infection or some other condition based on historical data (Allard, 1998). If such forecasting can be performed reliably, then the efficiency and effectiveness of public health systems can be enhanced (Groseclose and Buckeridge, 2017). Surveillance can be achieved using targeted sampling or through data that is already being collected for other purposes (Wagner et al., 2001). There are many potential data sources and diverse applications. Medical diagnostics devices are subject to quality control, and deviations from their designed behavior must be efficiently identified (Mitra, 2016). Hospitalized patients are constantly monitored in real time as part of their clinical care. With modern health monitoring systems, abnormalities in vital signs can immediately trigger response to such critical events as cardiac failure, respiratory complications, shock, or sepsis (Paganelli et al., 2022). Healthy or chronically ill individuals are tested at the clinic at regular intervals, and decisions to change their treatment regimen must be deduced from this data (Adlung et al., 2021).

In the context of pathogen control, syndromic surveillance considers the number of patients presenting with a constellation of symptoms. This data can be used to detect disease trends and outbreaks early and to inform if action is warranted (Reis and Mandl, 2003; Reis et al., 2007). Active surveillance for pathogens includes wastewater sampling, which tracks the presence and abundance of a pathogen over time in the local sewershed. Wastewater sampling has been successfully applied in surveillance for poliovirus (Asghar et al., 2014) and more recently for SARS-CoV-2 (Parkins et al., 2024), influenza (Dlamini et al., 2024), and many other pathogens (Boehm et al., 2023; Singh et al., 2024). Active surveillance also encompasses monitoring for zoonotic diseases, where the vector population is regularly trapped and analyzed. This approach is useful for quickly identifying and responding to localized outbreaks of numerous vector-borne diseases, such as West Nile fever (Petersen et al., 2013), eastern equine encephalitis (Armstrong and Andreadis, 2021), and Lyme disease (Eisen and Paddock, 2021). Examination of urbanization trends, human mobility patterns, and remote sensing data of environmental variables shows promise for forecasting the risk of other vector-borne diseases, including malaria and dengue (Kalluri et al., 2007; Pley et al., 2021). Public health organizations, such as the World Health Organization (WHO) and the Centers for Disease Control and Prevention (CDC), are building large databases and investing in substantial informatics infrastructure for intercepting infectious disease outbreaks (Geneva: World Health Organization and International Telecommunication Union, 2020; Centers for Disease Control and Prevention (U.S.). Office of Public Health Data, Surveillance, and Technology, 2023). Advancements in data science and machine learning have greatly expanded the potential for leveraging these systems to guide public health policy (Koplewitz et al., 2022; Stolerman et al., 2023).

For any scenario in which temporal surveillance is implemented, the purpose, therefore, is to use the signal to inform action (Kretzschmar, 2020; Mugahid et al., 2023; Lipsitch and Grad, 2024). If the time series indicates an abnormality in the sampled system that could lead to harm, then appropriate measures can be taken in response (Brooks et al., 2015; Flaxman et al., 2020; Pangallo et al., 2024). But how much deviation from some baseline is required to trigger an intervention (Teklehaimanot et al., 2004; Aminikhanghahi and Cook, 2017; Blazquez-Garcia et al., 2021)? If our threshold for acting is too high, then the condition or disease may spread before any countermeasures are taken, causing substantial harm. If our threshold is too low, then frequent overreactions result in accumulation of excessive intervention costs. The key point, then, is knowing when to intervene given a particular time-series signal.

## 2. Model

**Fig. 1.**
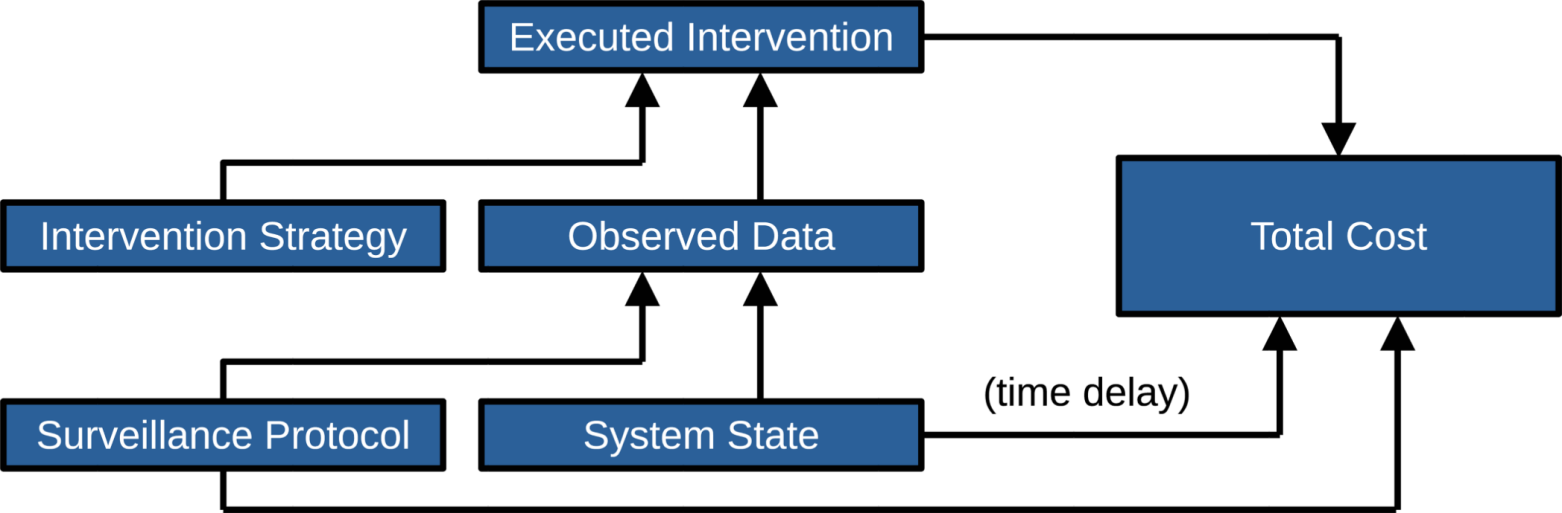
Building a surveillance and intervention platform. The system state affects both the total cost (after a time delay) and the observed data. The surveillance protocol affects both the total cost and the observed data. The observed data and the intervention strategy together determine whether an intervention is applied, and any executed intervention also contributes to the total cost.

We begin by identifying the basic components of the problem (Fig. 1). The system state could correspond to the functioning of a medical device, the condition of a patient, or the presence of an infectious agent in a population. The system state is not directly known, but its effects are observed (perhaps at a later date or time) and contribute to the total cost, unless we take immediate action. Therefore, we construct a surveillance protocol, which also contributes to the total cost (even if only negligibly) and produces a data stream that provides real-time information on the system state. We further specify a decision rule for whether to intervene based on this data. Whenever we intervene, there is a contribution to the total cost.

**Fig. 2.**
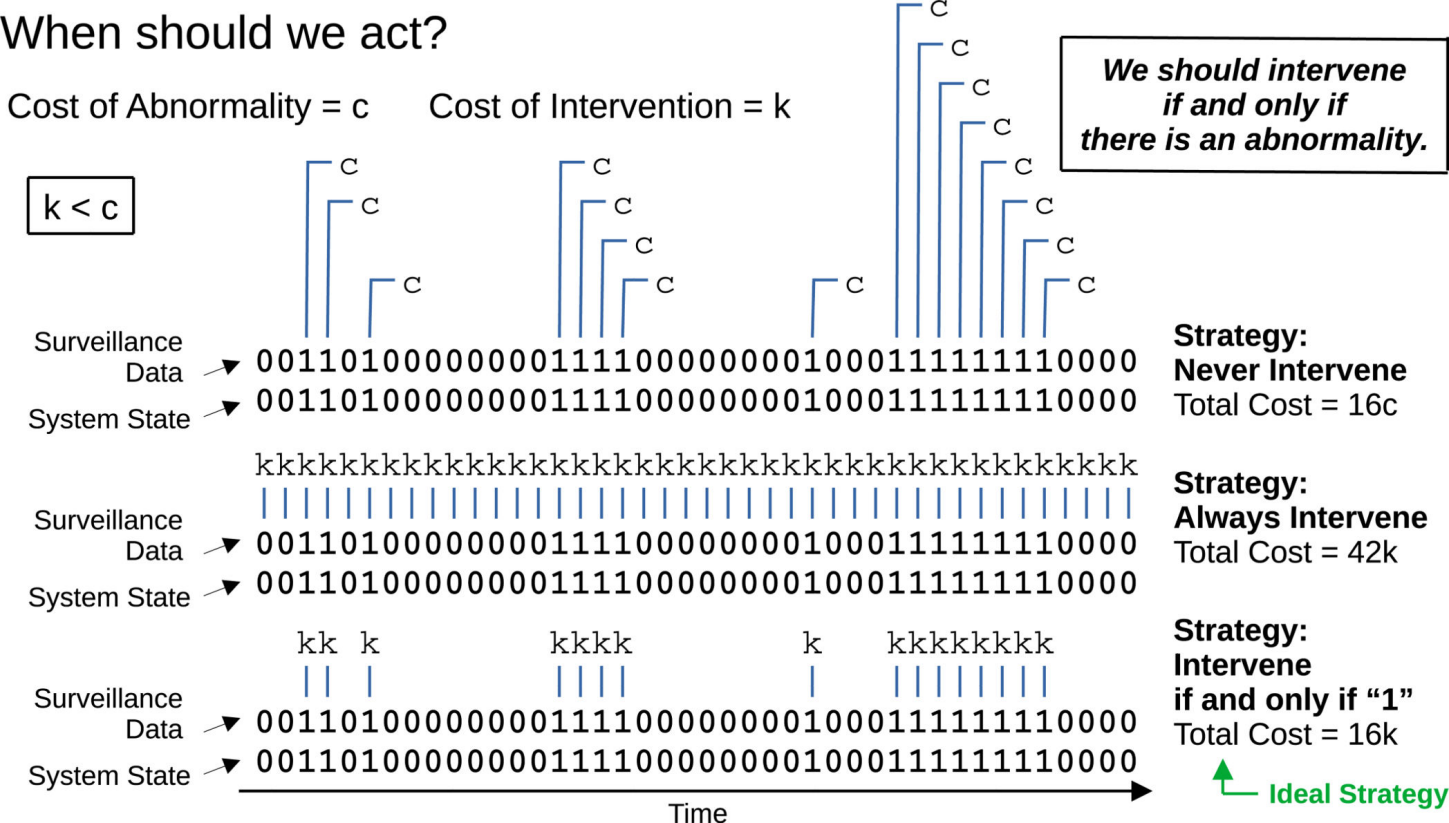
We would ideally intervene exactly when the system state is abnormal. At each time point, our system is in one of two states: normal or abnormal. If the system is normal, then there is no cost incurred related to any abnormality. If the system is abnormal and we do not intervene, then a cost, c, is incurred. Whenever we intervene, we always incur a cost, k. For k<c, the optimal strategy is to intervene if and only if there is an abnormality. (For generating the bit sequences, we set $q_{01}=0.1$, $q_{10}=0.3$, $p_{01}=0$, and $p_{10}=0$).

The system state, the surveillance protocol, and the intervention strategy therefore determine the total cost, and we must choose the surveillance protocol and intervention strategy that minimize this quantity (von Neumann and Morgenstern, 2004; Olejarz et al., 2024). To make progress, we treat each of the three contributions to the total cost in its simplest manifestation. First, consider the system surveyed, which could be the operational integrity of a medical device, a patient’s biological or physiological condition, or the state of an entire population of individuals. The state of the system could typically be classified using many variables. In our model, however, we assume that the system at any given time is in one of two possible states — normal or abnormal — and we treat time as being discrete. The state of the system versus time is therefore a binary time series (Kedem and Fokianos, 2002; Jentsch and Reichmann, 2019). If the system is in the abnormal state at a particular time, *t*, and no action is taken, then a resulting cost, *c*, is incurred. This could, for example, arise from operational failure of a diagnostics device, deterioration of a patient’s or population’s condition, or unmitigated spread of an infectious agent. If we take action at time *t*, then we instead incur a different cost, *k*. (We assume that any costs incurred at one time step have no effect on costs incurred at the next. An example would be the costs incurred due to a vector-borne pathogen for which humans are dead-end hosts. In this case, spillover infections (and their associated costs) during one time interval are independent of spillover infections during the next. Similarly, if people are advised to stay indoors, then the associated intervention costs are felt only during the time interval in which they are employed.) Since the two possible responses at each time point are either action or inaction, the decisions to intervene or to not intervene also form a binary time series. The optimization problem presents when *k < c*. (Another way to state this is that *k < c* defines *c* to be a cost associated with an abnormality, since if that inequality does not hold, intervention in our model is ineffective.) We want to intervene if and only if the system is in the abnormal state, thereby averting harm from all threats without accumulating any unnecessary intervention costs (Fig. 2).

To do that, we further suppose that the observed data stream is a binary time series—i.e., at each time point, our surveillance program reads and reports a single bit of data, reflecting the system state as either normal or abnormal. The meaning of this bit can vary. If a device is being tested for proper functioning, then the bit reflects its state of operation. If a patient is being monitored, then the bit reflects the concentration of a particular biomarker. If a population is being sampled, then the bit reflects the number of clinical cases or abundance of an infectious agent in a defined geographic area. (The bit might also be used as a guide for preemptive interventions. For example, global surveillance trends and patterns of regional interconnectedness might also be considered for anticipating spread of an infectious agent to the local population.) In all cases, there is a translation of a measured numeric value into a binary normal or abnormal indicator based on some threshold. Due to stochasticity, the imprecision of our measurement apparatus, and the imposition of our binary threshold, we neither directly nor accurately observe the state of the system, making our model similar to a hidden Markov model (Zucchini and MacDonald, 2009; Mor et al., 2021).

Hidden Markov models have been studied in diverse contexts and for many applications. They are valuable tools for the optimization of quality control, such as in fault detection (Smyth, 1994; Xu and Ge, 2004; Lee et al., 2010; Alshraideh and Runger, 2013), degradation assessment (Cholette and Djurdjanovic, 2014; Kisic et al., 2015; Zhang et al., 2016), predictive maintenance (Simoes et al., 2017; Martins et al., 2021), determination of water quality (Spezia et al., 2011; Li et al., 2022), and pharmaceutical process monitoring (Zhang et al., 2009). They have important uses in medical diagnostics surveillance, including analyzing changes in gene expression (Schliep et al., 2003), testing for drug effectiveness (Scott et al., 2005), checking cardiovascular function (Andreao et al., 2006), diagnosing respiratory illness (Matos et al., 2006), managing diabetes (Dicker et al., 2013), and screening for cancer (Meng et al., 2022). They have also been proposed for interpreting public health surveillance data (Le Strat and Carrat, 1999), with demonstrated uses in understanding nosocomial pathogen transmission (Cooper and Lipsitch, 2004), tuberculosis cases (Rafei et al., 2012), influenza epidemics (Martinez-Beneito et al., 2008; Conesa et al., 2015; Amoros et al., 2020), seasonal epidemics (Lin and Ludkovski, 2014; Lytras et al., 2018), COVID-19 data (Zhou et al., 2021), and other types of infectious disease outbreaks (McBryde et al., 2007; Watkins et al., 2009; Lu et al., 2010; Shmueli and Burkom, 2010; Zacher and Czogiel, 2022). Inspired by these applications, our work fills a critical void by assigning a cost to an abnormality, a cost per unit time to the surveillance protocol, and a cost to any executed intervention. This advancement precisely frames the optimization problem: Which surveillance protocol and intervention strategy results in the lowest expected cost per unit time?

Let $p_{00}$ and $p_{01}$ denote the probabilities of observing a 0 or 1, respectively, at time t given that the system is in the normal state at time t. Let $p_{10}$ and $p_{11}$ denote the probabilities of observing a 0 or 1, respectively, at time t given that the system is in the abnormal state at time t. Since our apparatus can only report a 0 or 1 at each measurement,

$$\begin{array}{c} p_{00}+p_{01}=1,\\ p_{10}+p_{11}=1. \end{array}$$

We assume these probabilities to be time-independent. Thus, the temporal data generated by our detector while the system is in a particular state is a realization of a Bernoulli process. We further consider that the abnormal state results in a higher probability of observing a 1 than the normal state:

$$p_{11}>p_{01}.$$

We postulate a simple rule for how the system can change. If the system is in the normal state at time t, then at time t+1, it is in the abnormal state with probability $q_{01}$ or the normal state with probability $q_{00}$. If the system is in the abnormal state at time *t*, then at time t+1, it is in the normal state with probability $q_{10}$ or the abnormal state with probability $q_{11}$. Since the state of the system at any given time is either normal or abnormal,

$$\begin{array}{c} q_{00}+q_{01}=1,\\ q_{10}+q_{11}=1. \end{array}$$

We assume these probabilities to be time-independent. Similarly, we assume that whenever we intervene, doing so does not alter the system state. (So, for example, we would not use this model for understanding when to impose a lockdown. We could, however, use this model for understanding when to recommend staying indoors to prevent infection with a vector-borne pathogen, particularly when humans are dead-end hosts. In this latter example, the intervention reduces the number of human infections, but the underlying dynamics of the pathogen in the vector population remain unperturbed.) Practically, for any particular application, these probabilities could be estimated from historical data or from independent modeling, which is beyond the scope of this work. We make one further assumption:

$$\begin{array}{c} q_{11}>q_{10},\\ q_{00}>q_{01}. \end{array}$$

Thus, the system is more likely to remain in its present state at the next time point than to change states. With this constraint, the optimization of surveillance using two or more bits of data is a problem in reinforcement learning (Sutton and Barto, 2018).

**Fig. 3.**
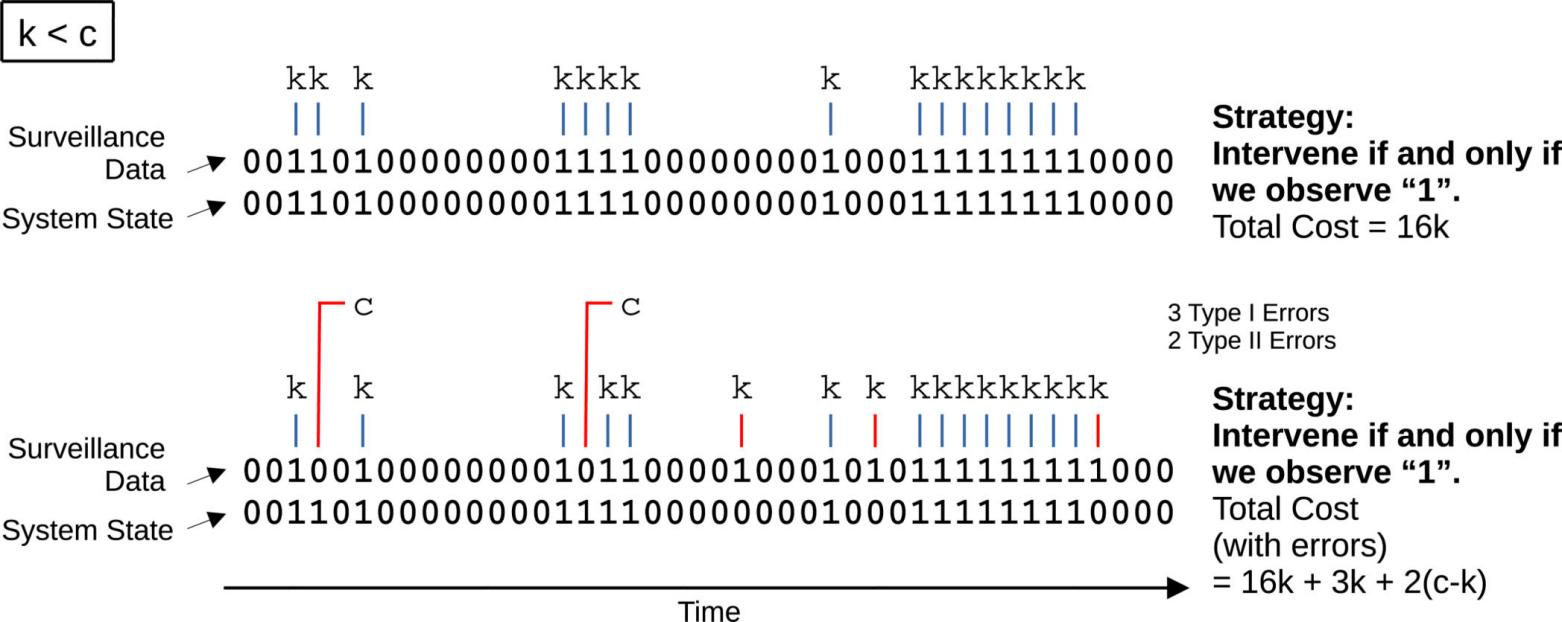
Accounting for measurement errors in the calculation of total cost. Whenever a Type I error occurs, we unnecessarily intervene and incur a cost, k. Whenever a Type II error occurs, we incur a cost, c, at a later time instead of having incurred a smaller, immediate cost, k, had we intervened. (For generating the bit sequences, we set $q_{01}=0.1$ and $q_{10}=0.3$. On top, we set $p_{01}=0$ and $p_{10}=0$, while on bottom, we set $p_{01}=0.1$ and $p_{10}=0.1$).

The task at hand is to use the resulting bit sequence to guide our intervention strategy. If $p_{00}=1$ and $p_{11}=1$, then we have exact knowledge of the state of the system, and the intervention strategy is to intervene if and only if we observe a 1. But such a scenario is an idealization. Realistically, we expect that $p_{00}<1$ and $p_{11}<1$—i.e., there are nonzero probabilities of both Type I and Type II errors (Figure S1). Whenever a Type I error occurs, we unnecessarily incur an intervention cost, k. Whenever a Type II error occurs, the delayed cost of inaction, c, exceeds the cost that we would have incurred had we intervened, k. We therefore want to minimize Type I and Type II errors (Fig. 3). How should we decide what to do in response to a noisy temporal signal?

For obtaining concrete results and building intuition, we make one further simplification. Consider $a_{1}(t)$, which is the probability that the system is in the abnormal state at a particular time, t. If the system is in the abnormal state at time t, then one of two things happened: Either the system was already in the abnormal state at time t−1 and did not transition to the normal state, or the system was in the normal state at time t−1 and transitioned to the abnormal state. This leads to the following recurrence relation:

(1) $$a_{1}(t)=a_{1}(t-1)\left[1-q_{10}\right]+\left[1-a_{1}(t-1)\right]q_{01}.$$

We simplify this by considering that the system is in steady state—

i.e., that the stochastic dynamics have run for sufficiently long that the probability that the system is in the abnormal state is time-independent. Substituting $a_{1}(t)\to a_{1}$ into Eq. (1) and solving for $a_{1}$, we obtain

(2) $$a_{1}=\frac{q_{01}}{q_{01}+q_{10}}.$$

Therefore, our prior — i.e., the probability that the system is in the abnormal state at any given time in the absence of any other information — is known exactly and is specified by Eq. (2). We also define $a_{0}$ to be the probability that the system is in the normal state at any given time, so that

$$a_{0}+a_{1}=1.$$

Bayesian inference thus yields the exact probability that the system is in the abnormal state given the most recent n bits of data (Gelman et al., 1995; Sarkka, 2013). By formally specifying a loss function that accounts for all possible types of costs and then minimizing this function, we can determine the ideal surveillance and intervention strategy.

All parameters are shown in Table 1.

## 3. Applications

To demonstrate the protocol, we first suppose that there is no surveillance. This serves as a baseline from which to gauge the effects of different surveillance and intervention strategies. We then explore how using a single bit of data from our detector to guide our intervention can be beneficial. We next explore the use of two bits of data from our detector, and we outline a methodology for evaluating progressively more complex surveillance and intervention protocols. For all calculations, we use the steady-state assumption of Eq. (2). We also consider all costs to be measured relative to the cost of an abnormality that is not acted on. Derivations of all analytical results are provided in the Supplementary Material.

### 3.1. No surveillance

Consider first the simplest possibility: that there is no surveillance at all. Let K=k/c denote the normalized intervention cost. If the system is typically in the normal state, or if K is unacceptably high, then intervening is too costly. But if there are frequent transitions to the abnormal state, or if K is sufficiently low, then intervening might be cost-effective. How should we decide what to do? If we always intervene, then the normalized cost incurred per unit time, which we denote $L_{0}(1)$, is equal to K. If we never intervene, then the expected normalized cost incurred per unit time, which we denote $L_{0}(0)$, is equal to $a_{1}$. Therefore, selecting between these two options, the optimal strategy is to always intervene if $a_{1}>K$ and never intervene otherwise. The intervention decision is thus solely informed by cost considerations.

### 3.2. Surveillance using one bit of data

We extend this framework to the next-simplest case: that we use the single most recent bit of data from our surveillance apparatus to inform our intervention strategy. Let $Y_{0}$ and $Y_{1}$ represent the intervention strategy given that the most recent bit is 0 or 1, respectively. $Y_{0}$ and $Y_{1}$ can each only take the values 0 or 1. If $Y_{0}=1$, then we always intervene when we observe a 0, and if $Y_{0}=0$, then we never intervene when we observe a 0. Likewise, if $Y_{1}=1$, then we always intervene when we observe a 1, and if $Y_{1}=0$ then we never intervene when we observe a 1. Let $S_{1}=s_{1}/c$ denote the normalized cost per unit time of implementing surveillance using one bit of data. The expected normalized cost per unit time is equal to

(3) $$L_{1}\left(Y_{0},Y_{1}\right)=a_{1}+S_{1}-\Delta_{0}Y_{0}-\Delta_{1}Y_{1}.$$

Here, as shorthand notation, we have defined

(4) $$\Delta_{i}=(1-K)\left(a_{1}p_{1i}\right)-K\left(a_{0}p_{0i}\right).$$

($\Delta_{i}$ is the expected normalized cost per unit time of not intervening when we observe i minus the expected normalized cost per unit time of intervening when we observe i.) The ideal intervention strategy when using one bit of data is the one for which $L_{1}\left(Y_{0},Y_{1}\right)$ is minimized. The consideration is whether $\Delta_{0}$ and $\Delta_{1}$, given by Eqs. (4), are positive or negative. From Eq. (3), if $\Delta_{0}>0$ or $\Delta_{0}<0$, then we should set $Y_{0}=1$ or $Y_{0}=0$, respectively. Likewise, if $\Delta_{1}>0$ or $\Delta_{1}<0$, then we should set $Y_{1}=1$ or $Y_{1}=0$, respectively. The optimal intervention strategy is then

(5) $$Y_{i}^{*}=\theta \left(\Delta_{i}\right).$$

Here, θ denotes the Heaviside step function. Substituting $Y_{i}=Y_{i}^{*}$, given by Eqs. (5), into Eq. (3), we minimize $L_{1}\left(Y_{0},Y_{1}\right)$.

**Fig. 4.**
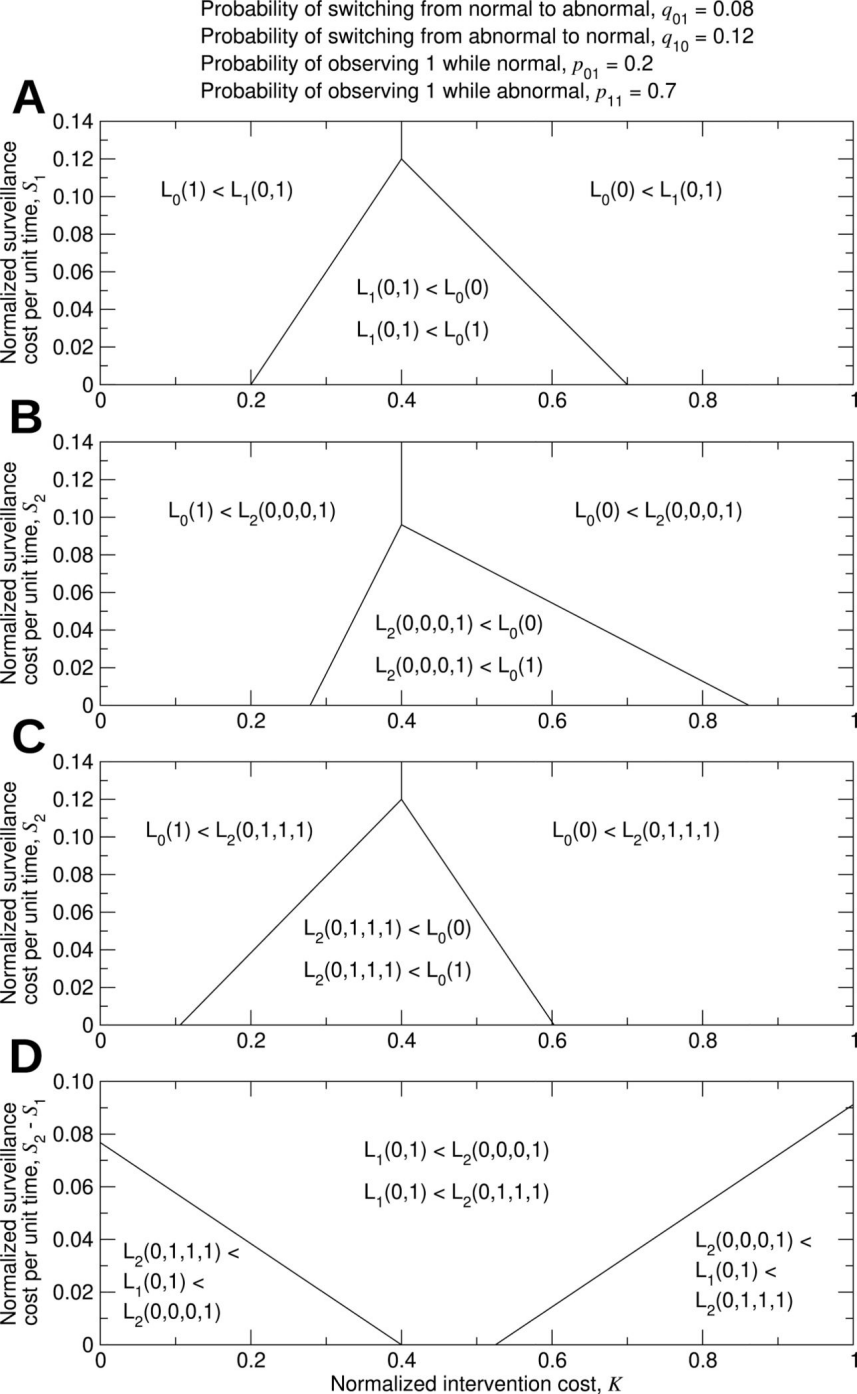
Surveillance using zero, one, or two bits of data. For this example, we consider five possibilities: (i) no surveillance; never intervene, (ii) no surveillance; always intervene, (iii) surveillance using one bit; intervene if and only if the most recent bit is 1, (iv) surveillance using two bits; intervene if and only if both of the two most recent bits are 1, or (v) surveillance using two bits; intervene if and only if at least one of the two most recent bits is 1. We compare strategies (i), (ii), and (iii) in (A), strategies (i), (ii), and (iv) in (B), strategies (i), (ii), and (v) in (C), and strategies (iii), (iv), and (v) in (D). The ideal surveillance and intervention strategy is the one for which the corresponding cost function among the five considered possibilities is lowest, which is determined by the values of K, $S_{1}$, and $S_{2}$.

Considering that we can use either no surveillance or surveillance based on the most recent bit, there are six possible strategies and six corresponding loss functions: $L_{0}(0)$, $L_{0}(1)$, $L_{1}(0,0)$, $L_{1}(1,0)$, $L_{1}(0,1)$, and $L_{1}(1,1)$. But the analysis simplifies. $L_{1}(0,1)<L_{1}(1,0)$ is necessarily true, since an observation of 1 in the most recent bit indicates a higher probability of being in the abnormal state. Also, $L_{0}(Y)<L_{1}(Y,Y)$ is necessarily true, since using the most recent bit of data entails a normalized surveillance cost per unit time of $S_{1}$, whereas using no data entails no surveillance cost. There are then three possibilities: We can choose to (i) never intervene, (ii) always intervene, or (iii) intervene if and only if the most recent bit is 1. We should choose the first option if $L_{0}(0)<L_{0}(1)$ and $L_{0}(0)<L_{1}(0,1)$. We should choose the second option if $L_{0}(1)<L_{0}(0)$ and $L_{0}(1)<L_{1}(0,1)$. We should choose the third option if $L_{1}(0,1)<L_{0}(0)$ and $L_{1}(0,1)<L_{0}(1)$. The numerical optimization problem using one bit of data is illustrated in Table S1. Optimization of surveillance using one bit of data is summarized in Fig. 4A.

The decision of whether to use surveillance based on the most recent bit of data depends on the values of K and $S_{1}$, and Fig. 4A splits the parameter space into three regions. In the region at right, we should not use surveillance, and we should never intervene. In the region at left, we should not use surveillance, and we should always intervene as standard practice. Surveillance should only be implemented within the triangular region.

A simple example helps for putting the model and its potential applications into context. Many vector-borne pathogens, such as West Nile virus and eastern equine encephalitis virus, persist in the environment and are characterized by sudden, intermittent outbreaks. The timing of these outbreaks is extremely difficult to predict. To model this, suppose that $q_{01}$ — defined for this example as the probability of an outbreak beginning in any given week — is small $\left(q_{01}\ll 1\right)$. It is also realistic to suppose that outbreaks do not suddenly and spontaneously go away $\left(q_{10}\ll 1\right)$.

Suppose that the vector population is sampled and tested weekly, and we measure the abundance of viral RNA, v, in each vector sample. When there is no outbreak, the abundance of viral RNA in each measurement is drawn from a probability distribution, $F_{0}(v)$. For each measurement during an ongoing outbreak, the abundance of viral RNA is drawn from a different probability distribution, $F_{1}(v)$. We define a cutoff value for the measured abundance of viral RNA in a sample, $v^{\prime }$. The distributions $F_{0}(v)$ and $F_{1}(v)$ are such that the probability of observing $v\geq v^{\prime }$ when there is no outbreak, $p_{01}$, is less than the11 probability of observing $v\geq v^{\prime }$ when there is an ongoing outbreak, $p_{11}$.

If $v<v^{\prime }$, then we interpret this as meaning that there is no outbreak, while if $v\geq v^{\prime }$, then we suspect an ongoing outbreak and take appropriate countermeasures. The countermeasures could take many forms. For example, standing water can be drained from containers to reduce the population of mosquito larvae, and large-scale spraying of pesticides can be carried out. These actions directly target the vector population, however, which means that they would necessarily raise the value of $q_{10}$ when applied. Since we assume $q_{10}$ to be constant in the present treatment, we do not consider these types of interventions for this example. Rather, we consider interventions that lessen the probability of an individual contracting the pathogen, such as advising people to stay indoors and to use insect repellent when they must go outside.

These countermeasures have associated costs and benefits. By remaining indoors due to a public health advisory, people are foregoing activities that they would have otherwise engaged in. The use of insect repellent requires a non-negligible amount of money and time. But by implementing these countermeasures, we reduce the number of human infections due to the outbreak, thereby lessening pathogen-related costs due to morbidity and mortality.

The final step, then, is to understand how the aforementioned costs and benefits manifest in the model parameters k and c. In this example, k measures the costs of the intervention, and we assume that the intervention is perfectly effective at preventing new human infections. By contrast, *c* measures the costs of the pathogen given that no intervention is applied. If we are able to reconcile all types of costs using a common cost unit, then K=k/c is a dimensionless number, and Eqs. (5) specify the ideal surveillance and intervention strategy, given a particular viral RNA threshold, $v^{\prime }$. But what value of $v^{\prime }$ minimizes $L_{1}\left(Y_{0},Y_{1}\right)$? This value of $v^{\prime }$, which we denote $v^{*}$, is the optimal viral RNA threshold for triggering intervention.

### 3.3. Surveillance using two bits of data

Extending this framework further, suppose that we use the two most recent bits of data from our surveillance apparatus to inform our intervention strategy. Let $Y_{ij}$ represent the intervention strategy given that the most recent bit is j and the bit before that is i. Each $Y_{ij}$ can only take the values 0 or 1. If $Y_{ij}=1$, then we always intervene when we observe ij, and if $Y_{ij}=0$, then we never intervene when we observe ij. Let $S_{2}=s_{2}/c$ denote the normalized cost per unit time of implementing surveillance using two bits of data. The expected normalized cost per unit time is equal to

(6) $$L_{2}\left(Y_{00},Y_{10},Y_{01},Y_{11}\right)=a_{1}+S_{2}-\Delta_{00}Y_{00}-\Delta_{10}Y_{10}-\Delta_{01}Y_{01}-\Delta_{11}Y_{11}.$$

Here, as shorthand notation, we have defined

(7) $$\Delta_{ij}=(1-K)\left(a_{1}p_{1i}q_{11}p_{1j}+a_{0}p_{0i}q_{01}p_{1j}\right)-K\left(a_{1}p_{1i}q_{10}p_{0j}+a_{0}p_{0i}q_{00}p_{0j}\right).$$

($\Delta_{ij}$ is the expected normalized cost per unit time of not intervening when we observe ij minus the expected normalized cost per unit time of intervening when we observe ij.) The ideal intervention strategy when using two bits of data is the one for which $L_{2}\left(Y_{00},Y_{10},Y_{01},Y_{11}\right)$ is minimized. The consideration is whether $\Delta_{ij}$, given by Eqs. (7), are positive or negative. From Eq. (6), if $\Delta_{ij}>0$ or $\Delta_{ij}<0$, then we should set $Y_{ij}=1$ or $Y_{ij}=0$, respectively. The optimal intervention strategy is then

(8) $$Y_{ij}^{*}=\theta \left(\Delta_{ij}\right).$$

Substituting $Y_{ij}=Y_{ij}^{*}$, given by Eqs. (8), into Eq. (6), we minimize $L_{2}\left(Y_{00},Y_{10},Y_{01},Y_{11}\right)$.

When the possibility of using two bits of data is considered, optimization of surveillance becomes a more involved calculation (Figures S2 and S3). To understand how to optimize surveillance using two bits of data, we must consider the values of K, $S_{1}$, and $S_{2}$. There are five possibilities: We can choose to (i) never intervene, (ii) always intervene, (iii) intervene if and only if the most recent bit is 1, (iv) intervene if and only if the two most recent bits are 1, or (v) intervene if and only if at least one of the two most recent bits is 1. For determining which surveillance and intervention strategy is best, there are thus five loss functions to consider: $L_{0}(0)$, $L_{0}(1)$, $L_{1}(0,1)$, $L_{2}(0,0,0,1)$, and $L_{2}(0,1,1,1)$. We should choose the first strategy if $L_{0}(0)$ is lowest, the second strategy if $L_{0}(1)$ is lowest, the third strategy if $L_{1}(0,1)$ is lowest, the fourth strategy if $L_{2}(0,0,0,1)$ is lowest, or the fifth strategy if $L_{2}(0,1,1,1)$ is lowest. The numerical optimization problem using two bits of data is illustrated in Table S2. Optimization of surveillance using two bits of data is summarized in Fig. 4.

**Fig. 5.**
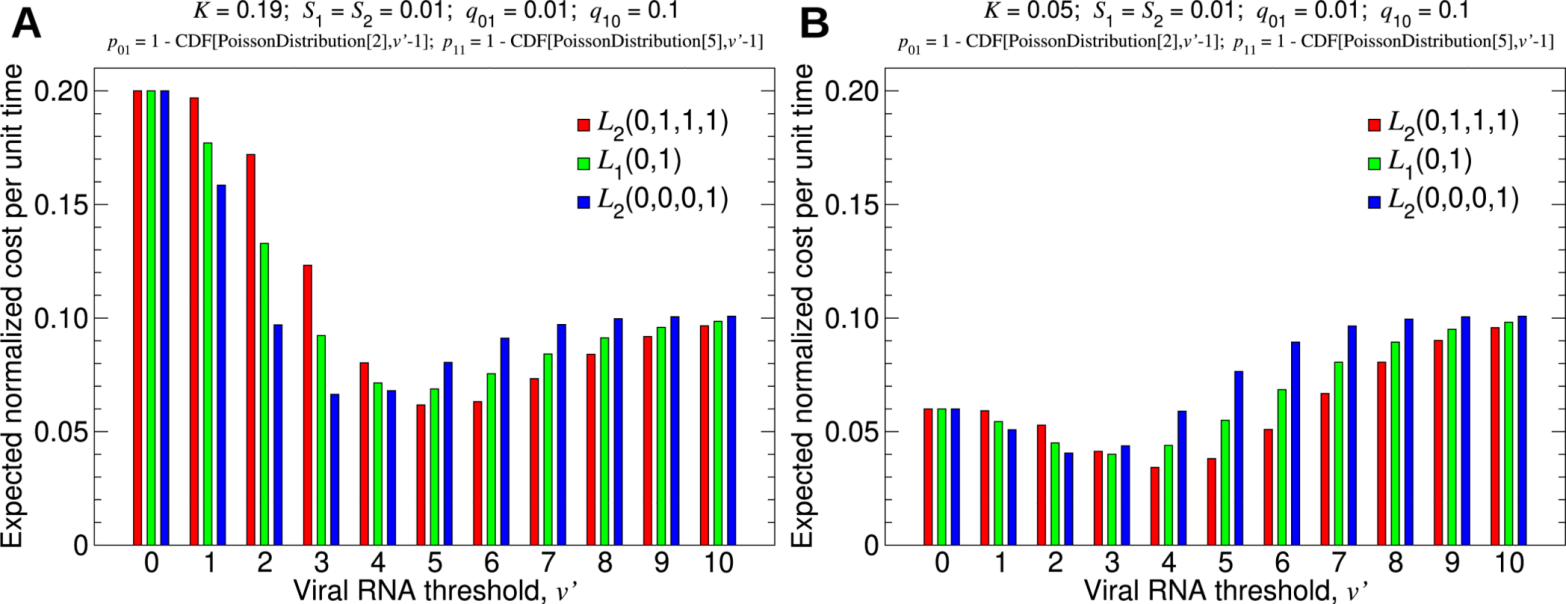
Choosing the optimal viral RNA threshold and the optimal number of bits to use. We consider three possible strategies: intervene if and only if the most recent bit is 1, intervene if and only if the two most recent bits are 1, and intervene if and only if at least one of the two most recent bits is 1. The corresponding loss functions are $L_{1}(0,1)$, $L_{2}(0,0,0,1)$, and $L_{2}(0,1,1,1)$, respectively. In both (A) and (B), the strategy to use only a single bit of data is outperformed by the strategy to intervene if and only if at least one of the two most recent bits is 1. In (A), we have $v^{*}=5$, and we should intervene if and only if at least one of the two most recent samples contained five or more mosquitoes that tested positive. In (B), we have $v^{*}=4$, and we should intervene if and only if at least one of the two most recent samples contained four or more mosquitoes that tested positive.

As an example of using two bits of data, let us return to the problem of optimizing environmental surveillance for arboviruses. Using two measurements in succession, how should we decide whether to intervene? It is worth noting that a proper decision rule in this setting would use all available information, which would include the viral RNA abundance measured in the samples from both the previous week and the current week. But for applying this model, we use only a single bit of data to represent each weekly measurement. If $v\geq v^{\prime }$ for the previous week, then the previous week’s observation is 1, and 0 otherwise. If $v\geq v^{\prime }$ for the current week, then the current week’s observation is 1, and 0 otherwise. Given a particular viral RNA threshold, $v^{\prime }$, Eqs. (8) determine the ideal surveillance and intervention strategy, and we must choose the optimal value of $v^{\prime }$, given by $v^{*}$, such that $L_{2}\left(Y_{00},Y_{10},Y_{01},Y_{11}\right)$ is minimized. A simple demonstration considering strategies that use either one or two bits of data is shown in Fig. 5.

### 3.4. Surveillance using any number of bits of data

The steps in the calculations are the same if we use any number of bits, n, for surveillance. Let $Y_{\left(b_{i}\right)}$ represent the intervention strategy given that the most recent sequence of bits is ${\left(b_{i}\right)}_{i=1}^{n}$. Each $Y_{\left(b_{i}\right)}$ can only take the values 0 or 1. If $Y_{\left(b_{i}\right)}=1$, then we always intervene when we observe ${\left(b_{i}\right)}_{i=1}^{n}$, and if $Y_{\left(b_{i}\right)}=0$, then we never intervene when we observe ${\left(b_{i}\right)}_{i=1}^{n}$. Let $S_{n}=s_{n}/c$ denote the normalized cost per unit time of implementing surveillance using n bits of data. For n≥1, the expected normalized cost per unit time is equal to

(9) $$L_{n}(\{Y\})=a_{1}+S_{n}-\sum_{{\left(b_{i}\right)}_{i=1}^{n}\forall b_{i}\in \{0,1\}}\Delta_{\left(b_{i}\right)}Y_{\left(b_{i}\right)}.$$

Here, as shorthand notation, we have defined

(10) $$\Delta_{\left(b_{i}\right)}=\sum_{{\left(r_{i}\right)}_{i=1}^{n}\forall r_{i}\in \{0,1\}}a_{r_{1}}p_{r_{1}b_{1}}\left(\prod_{i=2}^{n}q_{r_{i-1}r_{i}}p_{r_{i}b_{i}}\right)\left[(1-K)\delta_{r_{n}1}-K\delta_{r_{n}0}\right].$$

In Eqs. (10), $\delta_{ij}$ denotes the Kronecker delta. The ideal intervention strategy when using n bits of data is the one for which $L_{n}(\{Y\})$ is minimized. The consideration is whether each $\Delta_{\left(b_{i}\right)}$, given by Eqs. (10), is positive or negative. From Eq. (9), if $\Delta_{\left(b_{i}\right)}>0$ or $\Delta_{\left(b_{i}\right)}<0$, then we should set $Y_{\left(b_{i}\right)}=1$ or $Y_{\left(b_{i}\right)}=0$, respectively. If we use $Y_{\left(b_{i}\right)}^{*}$ to represent the optimal intervention strategy given that the n most recent bits are ${\left(b_{i}\right)}_{i=1}^{n}$, then we have

(11) $$Y_{\left(b_{i}\right)}^{*}\;=\;\theta \left(\Delta_{\left(b_{i}\right)}\right).$$

Substituting $Y_{\left(b_{i}\right)}=Y_{\left(b_{i}\right)}^{*}$, given by Eqs. (11), into Eq. (9), we minimize $L_{n}(\{Y\})$.

There are many possible scenarios to consider. For example, suppose that we are using the three most recent bits of data to inform our intervention strategy. If the three most recent bits are 001, then does the most recent observation of 1 outweigh the two prior observations of 00, or vice versa? If the three most recent bits are 110, then does the most recent observation of 0 outweigh the two prior observations of 11, or vice versa? Figure S4 shows that the answers to these questions are subtle and dependent on parameter values. We might also ask if using three bits of data in the decision to intervene is justified. For certain parameter values, surveillance using three bits of data delivers a substantial reduction in the expected normalized cost per unit time versus using only two bits of data (Figure S5). Optimizing surveillance using more than two bits of data must therefore be done carefully.

## 4. Discussion

In this work, the complexities of the system being monitored, the diagnostic measurements of that system, and the set of possible interventions are cleanly abstracted into a basic, operable theoretical framework. The key considerations behind optimizing surveillance and intervention are precisely formulated, and the parameter space can be exhaustively explored. While this approach is valuable as a conceptual aid, it also presents limitations and challenges for precisely modeling real-world surveillance problems. Yet, for many realistic settings, careful interpretation of the model parameters is all that is necessary for meaningful application of the model, as shown in the example of optimizing surveillance for arboviruses. In other settings, minimal extensions to the modeling approaches herein vastly expand their potential applicability.

Although our model is simple, it is broadly adaptable. Our assumption that the system state is binary is a valid first step in many settings. For example, a piece of equipment might normally operate around a stable equilibrium point (system state 0) and only begin to malfunction after a large perturbation forces it toward another stable equilibrium point (system state 1). A patient might be healthy for a long period (system state 0) before their condition suddenly worsens (system state 1). Or an infectious agent might be absent from a population for a long period (system state 0) and then spread rapidly following its introduction (system state 1). Our assumption that each measurement is binary could reflect a diagnostics device that only outputs two possible values, or it could result from dichotomizing a count variable using a suitable smoothing or preprocessing routine (Nelson et al., 2017). It could also reflect an oversimplification dictated by the realities of using data from a highly regulated industry. Our assumption that the intervention strategy is binary reflects the simplest possible type of intervention and is a reasonable starting point for any such analysis. The interpretation of time in the model can be adapted to the particular application at hand. For example, for reporting the prevalence of an infectious disease, each observation might reflect the number of new cases in a given week. The cost parameters are also adaptable and can include a wide variety of effects.

For this type of mathematical optimization to be effective, a suitable cost function to be minimized must be constructed. This is challenging, particularly since the system state and data reporting are ever-changing, complex processes that would not be reliably described by a small set of transition rules and parameters. A realistic implementation of this approach would be adaptive, requiring both inference of the rules governing the underlying process and prediction of an abnormality using the same data stream (Birrell et al., 2020; Russell and Norvig, 2020; Lipsitch et al., 2024). A further challenge is that the most harmful threats often appear stochastically and at irregular intervals. This could be handled by including an additional cost parameter that is necessarily larger when using an intervention strategy that is less aggressive. This generates an incentive to be preemptive in reacting to abnormalities in the data—i.e., we tolerate higher intervention costs in exchange for the lessened probability of being devastated by a disturbance that grows uncontrollably.

Complicating this further is the variety of types of costs at play. If quality control is being performed on a device or manufacturing process, then intervention might entail fixing components or altering specifications that are provided to customers. If a patient is tested at the clinic, then intervention might mean changing the patient’s prescribed medications or suggesting modifications to their lifestyle. If an infectious disease is spreading, then intervention might involve imposing quarantines, encouraging masking, or developing vaccines. Although understanding these myriad costs using the same cost unit (e.g., time, money, effort, mental well-being, physical well-being, lives lost, etc.) seems daunting, determination of the optimal surveillance and intervention strategy requires it, since the parameters $S_{n}$ and K must be dimensionless. The necessity of a unified understanding of costs persists as more complex and realistic models of this sort are investigated.

Even with the uncertainties inherent in assignment of costs, our model is critical for guiding public health policy. Regardless of rationale or justification, a practitioner necessarily believes that the surveillance and intervention protocol that they are using is optimal. When conditions change — e.g., if more people are presenting to the hospital, or if an arbovirus’s prevalence in the environment is increasing — a corresponding alteration to the surveillance and intervention program is warranted. Our model accordingly enables relative comparisons between strategies given different assumptions, and definitive recommendations to the public health practitioner on adaptations that should be made can be offered. In a similar vein, surveillance and intervention strategies differ geographically, again based on different location-specific observations and assumptions. If a particular policy is believed to be optimal for one location, then, based on different observations and assumptions, our model suggests suitable changes that should be made for another location.

Our methodology can be further developed and applied to complex real-world scenarios, where the state of the system is not binary. For example, equipment malfunctions can progress through stages such as warnings or partial functionality before full failure. Similarly, a patient’s health condition could range from mild to moderate to severe, and disease spread often involves multiple stages, such as latent, early spread, exponential, or stabilization. In a natural extension of this framework, one might accordingly consider that after a disturbance is initiated, its magnitude grows in time according to a branching process (Harris, 1963; Athreya and Ney, 1972). If we wait longer before responding to the abnormality, then we incur a larger cost due to its effects. One might further suppose that our detector outputs a count at each time point, and we must use this data to inform our belief of whether a disturbance is present and whether to intervene. The optimization problem works the same: If our threshold for acting is too low, then the small expected size of an abnormality when we intervene does not justify the frequent intervention costs that are incurred. If our threshold for acting is too high, then an abnormality would typically grow to a large size before intervention is applied, and the intervention would only have minimal effectiveness. There might also be multiple disturbances of various magnitudes overlapping with each other, which could increase the chances of Type I and Type II errors. The task is therefore to determine whether we should intervene given a particular sequence of recent count measurements. This is an important direction for future work.

We have constructed a simple model for time-series surveillance and investigated its implications. A salient point is that surveillance can be either beneficial or detrimental, depending on many factors. Proposed applications of surveillance must be properly vetted for their usefulness before substantial resources are allocated to their development. Once a surveillance system is built, it is not enough to be able to identify or predict anomalies: Any observed signal must be integrated within a cost-benefit framework for understanding the consequences of particular responses to surveillance data. The surveillance system can only be meaningfully optimized by constructing a suitable cost function — with all cost parameters interpreted using a common cost unit — and identifying the intervention strategy for which this function is minimal. Even if precise assignment of costs is difficult, our model facilitates critical comparisons between strategies based on differing assumptions. Our model is a robust prototype for the effective implementation of surveillance programs that are used to inform public health policy.

## Supplementary Material

1

## Figures and Tables

**Table 1:** Parameters of the model.

Basic Parameters	
$p_{00}$	Probability of observing 0 while in the normal state
$p_{01}$	Probability of observing 1 while in the normal state
$p_{10}$	Probability of observing 0 while in the abnormal state
$p_{11}$	Probability of observing 1 while in the abnormal state
$q_{00}$	Probability of remaining in the normal state
$q_{01}$	Probability of switching from the normal state to the abnormal state
$q_{11}$	Probability of remaining in the abnormal state
$q_{10}$	Probability of switching from the abnormal state to the normal state
c	Cost due to an abnormality that is not acted on
k	Intervention cost
$s_{n}$	Surveillance cost per unit time using *n* bits of data
Derived Parameters	
$a_{0}=q_{10}/\left(q_{10}+q_{01}\right)$	Exact prior probability of being in the normal state
$a_{1}=q_{01}/\left(q_{10}+q_{01}\right)$	Exact prior probability of being in the abnormal state
K=k/c	Normalized intervention cost
$S_{n}=s_{n}/c$	Normalized surveillance cost per unit time using *n* bits of data

## Data Availability

No data was used for the research described in the article.

## Appendix A. Supplementary data

All calculations are provided in the supplementary material file.

Supplementary material related to this article can be found online at https://doi.org/10.1016/j.epidem.2025.100866.
