# Supplementary material for: A binary prototype for time-series surveillance and intervention
Source: Epidemics. Author manuscript; Available in PMC 2026 Mar 30. (PMC13035050; doi:10.1016/j.epidem.2025.100866)
Supplement: 1 [file NIHMS2130206-supplement-1.pdf]

Supplementary Material:
A Binary Prototype for Time-Series Surveillance and Intervention

Jason Olejarz, Till Hoffmann, Alex Zapf, Douaa Mugahid,
Ross Molinaro, Chadwick Brown, Artem Boltyenkov, Taras Dudykevych,
Ankit Gupta, Marc Lipsitch, Rifat Atun, Jukka-Pekka Onnela,
Sarah Fortune, Rangarajan Sampath, Yonatan H. Grad

This Supplementary Material is organized as follows. In Section 1, we derive the optimal intervention strategy given that there is no surveillance. In Section 2, we derive the optimal surveillance and intervention strategy using the most recent bit of data. In Section 3, we derive the optimal surveillance and intervention strategy using the two most recent bits of data. In Section 4, we demonstrate some challenges when optimizing surveillance using more than two bits of data. In Section 5, the code for generating the bit sequences in Figures 2, 3, S1, S2, and S3 is provided.

1 No surveillance

If there is no surveillance, then the optimal strategy is either to never intervene or to always intervene. If we never intervene, then we incur an expected cost per unit time equal to $a_1 c$. If we always intervene, then we incur a cost per unit time equal to k. Let Y represent the intervention strategy, so that $Y = 0$ if we never intervene and $Y = 1$ if we always intervene. The unnormalized expected cost per unit time, $L_0'(k;Y)$, can be expressed as

$$L_0'(k;Y) = a_1(1-Y)c + (a_1 + a_0)Yk. \tag{S1}$$

Letting $K = k/c$ and $L_0(K;Y) = L_0'(k;Y)/c$ in Equation (S1), we obtain the normalized expected cost per unit time:

$$L_0(K;Y) = a_1(1-Y) + (a_1 + a_0)YK. \tag{S2}$$

Using $a_1 + a_0 = 1$ in Equation (S2) and simplifying, we obtain

$$L_0(K;Y) = a_1 - (a_1 - K)Y. \tag{S3}$$

Equation (S3) serves as a baseline for gauging the effectiveness of more complex surveillance and intervention strategies.

2 Surveillance using one bit of data

The unnormalized expected cost per unit time given that we use the single most recent bit of data to inform our intervention strategy, $L_1'(s_1, k; Y_0, Y_1)$, can be expressed as

$$L_1'(s_1, k; Y_0, Y_1) = s_1 + \sum_{i \in \{0,1\}} [(a_1 p_{1i})(1 - Y_i)c + (a_1 p_{1i} + a_0 p_{0i})Y_i k]. \tag{S4}$$

The terms in the summation in Equation (S4) have a simple interpretation. If we do not intervene at time t and the system is in the abnormal state at time t, then we incur a delayed cost, c. Considering the most recent bit of data, there are two ways that this can happen:

- The system can be in the abnormal state at time t, be measured as bit i at time t, and not be acted on at time t. This sequence of events occurs with probability $a_1 p_{1i}(1-Y_i)$, and i can be either 0 or 1.

If we intervene at time t, then we incur an immediate cost, k. Considering the most recent bit of data, there are four ways that this can happen:

- The system can be in the abnormal state at time t, be measured as bit i at time t, and be acted on at time t. This sequence of events occurs with probability $a_1 p_{1i} Y_i$, and i can be either 0 or 1.

- The system can be in the normal state at time t, be measured as bit i at time t, and be acted on at time t. This sequence of events occurs with probability $a_0 p_{0i} Y_i$, and i can be either 0 or 1.

Letting $K = k/c$, $S_1 = s_1/c$, and $L_1(S_1, K; Y_0, Y_1) = L_1'(s_1, k; Y_0, Y_1)/c$ in Equation (S4), we obtain the normalized expected cost per unit time:

$$L_1(S_1, K; Y_0, Y_1) = S_1 + \sum_{i \in \{0,1\}} [(a_1 p_{1i})(1 - Y_i) + (a_1 p_{1i} + a_0 p_{0i})Y_i K]. \tag{S5}$$

As shorthand notation, we define

$$\Delta_i = (1 - K)(a_1 p_{1i}) - K(a_0 p_{0i}). \tag{S6}$$

Rearranging Equation (S5), and using Equations (S6), we obtain

$$L_1(S_1, K; Y_0, Y_1) = a_1 + S_1 - \Delta_0 Y_0 - \Delta_1 Y_1. \tag{S7}$$

The task at hand is to use Equation (S7) and the observed bit sequence to guide our intervention strategy.

If $p_{01} = 0$ and $p_{11} = 1$, then we have exact knowledge of the state of the system, and the ideal intervention strategy is to intervene if and only if we observe a 1. But such a scenario is an idealization. Realistically, we expect that $p_{01} > 0$ and $p_{11} < 1$—i.e., there are nonzero probabilities of both Type I and Type II errors (Figure S1). Whenever a Type I error occurs, we unnecessarily incur an intervention cost, k. Whenever a Type II error occurs, the delayed cost of inaction, c, exceeds the immediate cost that we would have incurred had

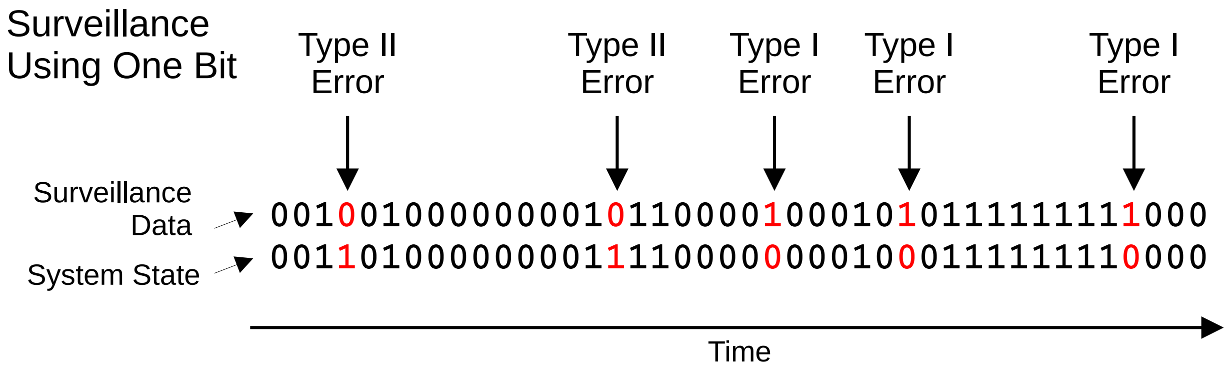

Figure S1: **Measurement errors.** Suppose that our intervention strategy is based on using only the most recent bit of data. A Type I error occurs whenever the system state is 0 and our diagnostics machine reports 1. A Type II error occurs whenever the system state is 1 and our diagnostics machine reports 0. (For generating the bit sequences, we set $q_{01} = 0.1$, $q_{10} = 0.3$, $p_{01} = 0.1$, and $p_{10} = 0.1$.)

we intervened, k. We therefore want to minimize Type I and Type II errors (Figure 3). A numerical example of optimizing surveillance using one bit of data is given in Table S1. How should we decide what to do in response to a noisy temporal signal?

It is helpful to understand the ideal surveillance and intervention strategy depending on the values of K and S_1 (Figure 4A). If K is larger than a certain value, which we denote K_+, then we should never intervene. K_+ is given by solving $L_1(0, K_+; 0, 1) = L_0(K_+; 0)$. We find

$$K_+ = \left(1 + \frac{q_{10}p_{01}}{q_{01}p_{11}}\right)^{-1}. \tag{S8}$$

If $K > K_+$ from Equation (S8), then the expected cost from reacting to false positives exceeds any benefit from averting the threat. The conclusion is that surveillance should not be executed, and intervention should never occur.

Similarly, if K is smaller than a certain value, which we denote K_-, then we should always intervene. K_- is given by solving $L_1(0, K_-; 0, 1) = L_0(K_-; 1)$. We find

$$K_- = \left(1 + \frac{q_{10}p_{00}}{q_{01}p_{10}}\right)^{-1}. \tag{S9}$$

If $K < K_-$ from Equation (S9), then the expected cost from dismissing false negatives exceeds any benefit from not intervening when there is no threat. The conclusion is that surveillance should not be executed, and intervention should always occur.

K_+ and K_-, given by Equations (S8) and (S9), respectively, are therefore key quantities for determining if surveillance is beneficial. If $K_- < K < K_+$, then surveillance might be justified if surveillance costs are sufficiently low. If we use surveillance, then we incur an expected cost per unit time equal to $L_1(S_1, K; 0, 1)$. First, consider that $K > a_1$. If we don't use surveillance, then the optimal strategy is to never intervene, and we incur an expected cost per unit time equal to $L_0(K; 0)$. We solve for the value of $S_1 = S_+$ for which

	$K = 0.1$	$K = 0.3$	$K = 0.4$	$K = 0.5$	$K = 0.8$
$Y = 0$	$L_0 = 0.40$	$L_0 = 0.40$	$L_0 = 0.40$	$\mathbf{L_0 = 0.40}$	$\mathbf{L_0 = 0.40}$
$Y = 1$	$\mathbf{L_0 = 0.10}$	$\mathbf{L_0 = 0.30}$	$L_0 = 0.40$	$L_0 = 0.50$	$L_0 = 0.80$
$Y_0, Y_1 = 0, 0$	$L_1 = 0.50$	$L_1 = 0.50$	$L_1 = 0.50$	$L_1 = 0.50$	$L_1 = 0.50$
$Y_0, Y_1 = 1, 0$	$L_1 = 0.44$	$L_1 = 0.56$	$L_1 = 0.62$	$L_1 = 0.68$	$L_1 = 0.86$
$Y_0, Y_1 = 0, 1$	$L_1 = 0.26$	$L_1 = 0.34$	$\mathbf{L_1 = 0.38}$	$L_1 = 0.42$	$L_1 = 0.54$
$Y_0, Y_1 = 1, 1$	$L_1 = 0.20$	$L_1 = 0.40$	$L_1 = 0.50$	$L_1 = 0.60$	$L_1 = 0.90$

Table S1: **Example of optimizing surveillance and intervention using the most recent bit of data.** We set $a_1 = 0.4$, $p_{01} = 0.2$, $p_{11} = 0.7$, and $S_1 = 0.1$.

$L_0(K; 0) = L_1(S_+, K; 0, 1)$:

$$S_+ = \Delta_1. \tag{S10}$$

If $S_1 < S_+$, then the benefit of appropriately intervening outweighs the cost of surveillance, and surveillance is beneficial. If $S_1 > S_+$, then surveillance is too expensive and is detrimental. From Equation (S10), S_+ is plotted versus K as the upper right boundary of the triangular region in Figure 4A.

Next, consider that $K < a_1$. If we don't use surveillance, then the optimal strategy is to always intervene, and we incur an expected cost per unit time equal to $L_0(K; 1)$. We solve for the value of $S_1 = S_-$ for which $L_0(K; 1) = L_1(S_-, K; 0, 1)$:

$$S_- = K - a_1 + \Delta_1. \tag{S11}$$

If $S_1 < S_-$, then the benefit of appropriately intervening outweighs the cost of surveillance, and surveillance is beneficial. If $S_1 > S_-$, then surveillance is too expensive and is detrimental. From Equation (S11), S_- is plotted versus K as the upper left boundary of the triangular region in Figure 4A.

3 Surveillance using two bits of data

The unnormalized expected cost per unit time given that we use the two most recent bits of data, $L'_2(s_2, k; Y_{00}, Y_{10}, Y_{01}, Y_{11})$, can be expressed as

$$\begin{aligned} &L'_2(s_2, k; Y_{00}, Y_{10}, Y_{01}, Y_{11}) = s_2 \\ &+ \sum_{i,j \in \{0,1\}} [(a_1 p_{1i} q_{11} p_{1j} + a_0 p_{0i} q_{01} p_{1j})(1 - Y_{ij})c \\ &+ (a_1 p_{1i} q_{11} p_{1j} + a_0 p_{0i} q_{01} p_{1j} + a_0 p_{0i} q_{00} p_{0j} + a_1 p_{1i} q_{10} p_{0j}) Y_{ij} k]. \end{aligned} \tag{S12}$$

The terms in the summation in Equation (S12) have a simple interpretation. If we do not intervene at time t and the system is in the abnormal state at time t, then we incur a delayed cost, c. Considering the two most recent bits of data, there are eight ways that this can happen:

- The system can be in the abnormal state at time $t - 1$, be measured as bit i at time $t-1$, remain in the abnormal state at time t, be measured as bit j at time t, and not be

acted on at time t. This sequence of events occurs with probability $a_1 p_{1i} q_{11} p_{1j}(1-Y_{ij})$, and i and j can each be either 0 or 1.

- The system can be in the normal state at time $t-1$, be measured as bit i at time $t-1$, transition to the abnormal state at time t, be measured as bit j at time t, and not be acted on at time t. This sequence of events occurs with probability $a_0 p_{0i} q_{01} p_{1j}(1-Y_{ij})$, and i and j can each be either 0 or 1.

If we intervene at time t, then we incur an immediate cost, k. Considering the two most recent bits of data, there are sixteen ways that this can happen:

- The system can be in the abnormal state at time $t-1$, be measured as bit i at time $t-1$, remain in the abnormal state at time t, be measured as bit j at time t, and be acted on at time t. This sequence of events occurs with probability $a_1 p_{1i} q_{11} p_{1j} Y_{ij}$, and i and j can each be either 0 or 1.

- The system can be in the normal state at time $t-1$, be measured as bit i at time $t-1$, transition to the abnormal state at time t, be measured as bit j at time t, and be acted on at time t. This sequence of events occurs with probability $a_0 p_{0i} q_{01} p_{1j} Y_{ij}$, and i and j can each be either 0 or 1.

- The system can be in the normal state at time $t-1$, be measured as bit i at time $t-1$, remain in the normal state at time t, be measured as bit j at time t, and be acted on at time t. This sequence of events occurs with probability $a_0 p_{0i} q_{00} p_{0j} Y_{ij}$, and i and j can each be either 0 or 1.

- The system can be in the abnormal state at time $t-1$, be measured as bit i at time $t-1$, transition to the normal state at time t, be measured as bit j at time t, and be acted on at time t. This sequence of events occurs with probability $a_1 p_{1i} q_{10} p_{0j} Y_{ij}$, and i and j can each be either 0 or 1.

Letting $K = k/c$, $S_2 = s_2/c$, and $L_2(S_2, K; Y_{00}, Y_{10}, Y_{01}, Y_{11}) = L_2'(s_2, k; Y_{00}, Y_{10}, Y_{01}, Y_{11})/c$ in Equation (S12), we obtain the normalized expected cost per unit time:

$$\begin{aligned} &L_2(S_2, K; Y_{00}, Y_{10}, Y_{01}, Y_{11}) = S_2 \\ &+ \sum_{i,j\in\{0,1\}} [(a_1 p_{1i} q_{11} p_{1j} + a_0 p_{0i} q_{01} p_{1j})(1 - Y_{ij}) \\ &+ (a_1 p_{1i} q_{11} p_{1j} + a_0 p_{0i} q_{01} p_{1j} + a_0 p_{0i} q_{00} p_{0j} + a_1 p_{1i} q_{10} p_{0j}) Y_{ij} K]. \end{aligned} \tag{S13}$$

As shorthand notation, we define

$$\Delta_{ij} = (1 - K)(a_1 p_{1i} q_{11} p_{1j} + a_0 p_{0i} q_{01} p_{1j}) - K(a_1 p_{1i} q_{10} p_{0j} + a_0 p_{0i} q_{00} p_{0j}). \tag{S14}$$

Rearranging Equation (S13), and using Equations (S14), we obtain

$$L_2(S_2, K; Y_{00}, Y_{10}, Y_{01}, Y_{11}) = a_1 + S_2 - \Delta_{00} Y_{00} - \Delta_{10} Y_{10} - \Delta_{01} Y_{01} - \Delta_{11} Y_{11}. \tag{S15}$$

The task at hand is to use Equation (S15) and the observed bit sequence to guide our intervention strategy.

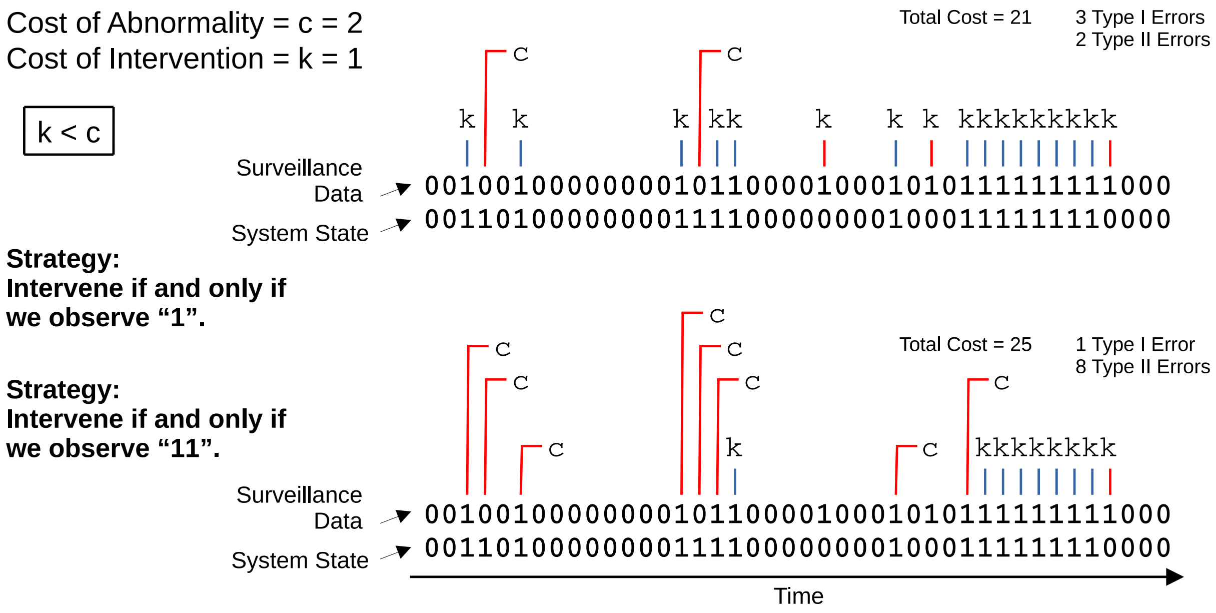

Figure S2: **Requiring reinforcement for intervening.** For this particular sequence of system state values and measurements, we compare the strategy to intervene if and only if we observe 1 versus the strategy to intervene if and only if we observe 11 in succession. For the latter strategy, there are two fewer Type I errors but six more Type II errors. The additional Type II errors mean that the latter strategy would have performed worse than the former for this realization of the dynamics. (For generating the bit sequences, we set $q_{01} = 0.1$, $q_{10} = 0.3$, $p_{01} = 0.1$, and $p_{10} = 0.1$.)

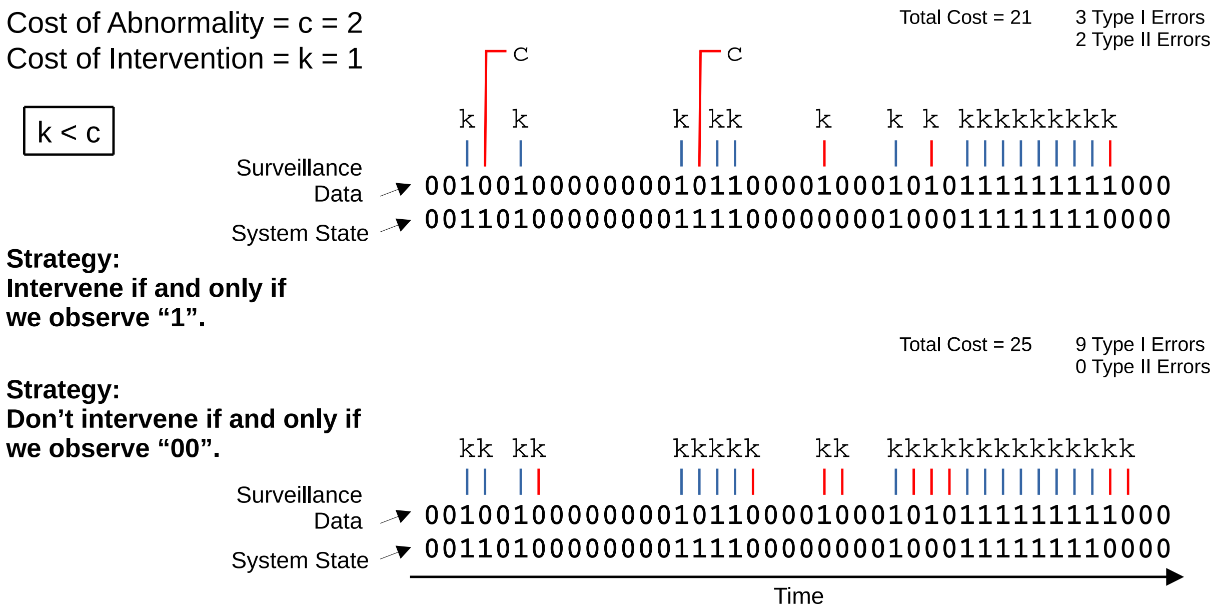

Figure S3: **Requiring reinforcement for not intervening.** For this particular sequence of system state values and measurements, we compare the strategy to intervene if and only if we observe 1 versus the strategy to not intervene if and only if we observe 00 in succession. For the latter strategy, there are two fewer Type II errors but six more Type I errors. The additional Type I errors mean that the latter strategy would have performed worse than the former for this realization of the dynamics. (For generating the bit sequences, we set $q_{01} = 0.1$, $q_{10} = 0.3$, $p_{01} = 0.1$, and $p_{10} = 0.1$.)

For some applications, surveillance using the two most recent bits of data can be detrimental. In Figure S2, for a particular sequence of system states and observed bits, we compare the strategy to intervene if and only if we observe 1 to the strategy to intervene if and only if we observe 11. For the latter strategy, the number of Type I errors is reduced but the number of Type II errors is substantially increased, such that requiring an observation of 11 in succession for deciding to intervene is counterproductive. In Figure S3, for the same sequence of system states and observed bits, we compare the strategy to intervene if and only if we observe 1 to the strategy to not intervene if and only if we observe 00. For the latter strategy, the number of Type II errors is reduced but the number of Type I errors is substantially increased, such that requiring an observation of 00 in succession for deciding to not intervene is counterproductive.

Another example shows how surveillance using the two most recent bits of data can be optimal (Table S2). If intervention costs are low, then it might be beneficial to not intervene if and only if both of the most recent bits are 0. By observing 00 in succession, we are more confident that the system is in the normal state and that intervention is useless. If intervention costs are high, then it might be beneficial to intervene if and only if both of the most recent bits are 1. By observing 11 in succession, we are more confident that the system is in the abnormal state and that intervention is worthwhile.

We have already worked out the conditions for when surveillance using one bit is superior to no surveillance. When we include the possibility of using two bits of data to guide our intervention, determination of the optimal surveillance and intervention strategy becomes more intricate. We must still consider how surveillance using one bit performs versus no surveillance, as shown in Figure 4A. We must similarly determine if surveillance using two bits is superior to no surveillance, and we must determine if surveillance using two bits is superior to surveillance using one bit.

When comparing surveillance using two bits to no surveillance, there are multiple comparisons that must be done separately. First, we compare the strategy to intervene if and only if we observe 11 to never intervening. If K is larger than a certain value, which we denote K_{0+}, then we should never intervene. K_{0+} is given by solving $L_2(0, K_{0+}; 0, 0, 0, 1) = L_0(K_{0+}; 0)$. We find

$$K_{0+} = \left[1 + \left(\frac{1}{K_+} - 1\right)\left(\frac{q_{00}p_{01} + q_{01}p_{11}}{q_{11}p_{11} + q_{10}p_{01}}\right)\right]^{-1}. \tag{S16}$$

If $K > K_{0+}$ from Equation (S16), then the expected cost from reacting to false positives whenever we observe 11 exceeds any benefit from averting the threat.

Similarly, we compare the strategy to intervene if and only if we observe 11 to always intervening. If K is smaller than a certain value, which we denote K_{0-}, then we should always intervene. K_{0-} is given by solving $L_2(0, K_{0-}; 0, 0, 0, 1) = L_0(K_{0-}; 1)$. We find

$$K_{0-} = \left[1 + \left(\frac{1}{K_-} - 1\right)\left(\frac{p_{00}p_{10} + p_{10}p_{01}(q_{00}p_{00} + q_{01}p_{10})}{p_{00}p_{10} + p_{00}p_{11}(q_{11}p_{10} + q_{10}p_{00})}\right)\right]^{-1}. \tag{S17}$$

If $K < K_{0-}$ from Equation (S17), then the expected cost from dismissing false negatives whenever we do not observe 11 exceeds any benefit from not intervening when there is no threat.

	$K = 0.3$	$K = 0.6$
$Y = 0$	$L_0 = 0.40000$	$L_0 = 0.40000$
$Y = 1$	$L_0 = 0.30000$	$L_0 = 0.60000$
$Y_0, Y_1 = 0, 0$	$L_1 = 0.40100$	$L_1 = 0.40100$
$Y_0, Y_1 = 1, 0$	$L_1 = 0.40500$	$L_1 = 0.60900$
$Y_0, Y_1 = 0, 1$	$L_1 = 0.29700$	$L_1 = 0.39300$
$Y_0, Y_1 = 1, 1$	$L_1 = 0.30100$	$L_1 = 0.60100$
$Y_{00}, Y_{10}, Y_{01}, Y_{11} = 0, 0, 0, 0$	$L_2 = 0.40200$	$L_2 = 0.40200$
$Y_{00}, Y_{10}, Y_{01}, Y_{11} = 1, 0, 0, 0$	$L_2 = 0.42596$	$L_2 = 0.56792$
$Y_{00}, Y_{10}, Y_{01}, Y_{11} = 0, 1, 0, 0$	$L_2 = 0.38204$	$L_2 = 0.44408$
$Y_{00}, Y_{10}, Y_{01}, Y_{11} = 1, 1, 0, 0$	$L_2 = 0.40600$	$L_2 = 0.61000$
$Y_{00}, Y_{10}, Y_{01}, Y_{11} = 0, 0, 1, 0$	$L_2 = 0.34604$	$L_2 = 0.40808$
$Y_{00}, Y_{10}, Y_{01}, Y_{11} = 1, 0, 1, 0$	$L_2 = 0.37000$	$L_2 = 0.57400$
$Y_{00}, Y_{10}, Y_{01}, Y_{11} = 0, 1, 1, 0$	$L_2 = 0.32608$	$L_2 = 0.45016$
$Y_{00}, Y_{10}, Y_{01}, Y_{11} = 1, 1, 1, 0$	$L_2 = 0.35004$	$L_2 = 0.61608$
$Y_{00}, Y_{10}, Y_{01}, Y_{11} = 0, 0, 0, 1$	$L_2 = 0.35396$	$\mathbf{L_2 = 0.38792}$
$Y_{00}, Y_{10}, Y_{01}, Y_{11} = 1, 0, 0, 1$	$L_2 = 0.37792$	$L_2 = 0.55384$
$Y_{00}, Y_{10}, Y_{01}, Y_{11} = 0, 1, 0, 1$	$L_2 = 0.33400$	$L_2 = 0.43000$
$Y_{00}, Y_{10}, Y_{01}, Y_{11} = 1, 1, 0, 1$	$L_2 = 0.35796$	$L_2 = 0.59592$
$Y_{00}, Y_{10}, Y_{01}, Y_{11} = 0, 0, 1, 1$	$L_2 = 0.29800$	$L_2 = 0.39400$
$Y_{00}, Y_{10}, Y_{01}, Y_{11} = 1, 0, 1, 1$	$L_2 = 0.32196$	$L_2 = 0.55992$
$Y_{00}, Y_{10}, Y_{01}, Y_{11} = 0, 1, 1, 1$	$\mathbf{L_2 = 0.27804}$	$L_2 = 0.43608$
$Y_{00}, Y_{10}, Y_{01}, Y_{11} = 1, 1, 1, 1$	$L_2 = 0.30200$	$L_2 = 0.60200$

Table S2: **Example of optimizing surveillance and intervention using the two most recent bits of data.** We set $q_{01} = 0.2$, $q_{10} = 0.3$, $p_{01} = 0.2$, $p_{11} = 0.5$, $S_1 = 0.001$, and $S_2 = 0.002$.

If $K_{0-} < K < K_{0+}$, then using two bits and intervening if and only if we observe 11 might be justified if surveillance costs are sufficiently low. If we use surveillance, then we incur an expected cost per unit time equal to $L_2(S_2, K; 0, 0, 0, 1)$. First, consider that $K > a_1$. If we don't use surveillance, then the optimal strategy is to never intervene, and we incur an expected cost per unit time equal to $L_0(K; 0)$. We set $L_0(K; 0) = L_2(S_{0+}, K; 0, 0, 0, 1)$ and solve for S_{0+}:

$$S_{0+} = \Delta_{11}. \tag{S18}$$

If $S_2 < S_{0+}$, then the benefit of appropriately intervening whenever we observe 11 outweighs the cost of surveillance, and surveillance using two bits is beneficial relative to never intervening. If $S_2 > S_{0+}$, then surveillance using two bits is too expensive relative to never intervening. From Equation (S18), S_{0+} is plotted versus K as the upper right boundary of the triangular region in Figure 4B.

Next, consider that $K < a_1$. If we don't use surveillance, then the optimal strategy is to always intervene, and we incur an expected cost per unit time equal to $L_0(K; 1)$. We set $L_0(K; 1) = L_2(S_{0-}, K; 0, 0, 0, 1)$ and solve for S_{0-}:

$$S_{0-} = K - a_1 + \Delta_{11}. \tag{S19}$$

If $S_2 < S_{0-}$, then the benefit of appropriately intervening whenever we observe 11 outweighs the cost of surveillance, and surveillance using two bits is beneficial relative to always intervening. If $S_2 > S_{0-}$, then surveillance using two bits is too expensive relative to always intervening. From Equation (S19), S_{0-} is plotted versus K as the upper left boundary of the triangular region in Figure 4B.

Next, we compare the strategy to not intervene if and only if we observe 00 to never intervening. If K is larger than a certain value, which we denote K_{1+}, then we should never intervene. K_{1+} is given by solving $L_2(0, K_{1+}; 0, 1, 1, 1) = L_0(K_{1+}; 0)$. We find

$$K_{1+} = \left[1 + \left(\frac{1}{K_+} - 1\right)\left(\frac{p_{11}p_{01} + p_{00}p_{11}(q_{00}p_{01} + q_{01}p_{11})}{p_{11}p_{01} + p_{10}p_{01}(q_{11}p_{11} + q_{10}p_{01})}\right)\right]^{-1}. \tag{S20}$$

If $K > K_{1+}$ from Equation (S20), then the expected cost from reacting to false positives whenever we do not observe 00 exceeds any benefit from averting the threat.

Similarly, we compare the strategy to not intervene if and only if we observe 00 to always intervening. If K is smaller than a certain value, which we denote K_{1-}, then we should always intervene. K_{1-} is given by solving $L_2(0, K_{1-}; 0, 1, 1, 1) = L_0(K_{1-}; 1)$. We find

$$K_{1-} = \left[1 + \left(\frac{1}{K_-} - 1\right)\left(\frac{q_{00}p_{00} + q_{01}p_{10}}{q_{11}p_{10} + q_{10}p_{00}}\right)\right]^{-1}. \tag{S21}$$

If $K < K_{1-}$ from Equation (S21), then the expected cost from dismissing false negatives whenever we observe 00 exceeds any benefit from not intervening when there is no threat.

If $K_{1-} < K < K_{1+}$, then using two bits and not intervening if and only if we observe 00 might be justified if surveillance costs are sufficiently low. If we use surveillance, then we incur an expected cost per unit time equal to $L_2(S_2, K; 0, 1, 1, 1)$. First, consider that $K > a_1$. If we don't use surveillance, then the optimal strategy is to never intervene, and we incur an

expected cost per unit time equal to $L_0(K;0)$. We set $L_0(K;0) = L_2(S_{1+}, K; 0, 1, 1, 1)$ and solve for S_{1+}:

$$S_{1+} = \Delta_{10} + \Delta_{01} + \Delta_{11}. \tag{S22}$$

If $S_2 < S_{1+}$, then the benefit of appropriately intervening whenever we do not observe 00 outweighs the cost of surveillance, and surveillance using two bits is beneficial relative to never intervening. If $S_2 > S_{1+}$, then surveillance using two bits is too expensive relative to never intervening. From Equation (S22), S_{1+} is plotted versus K as the upper right boundary of the triangular region in Figure 4C.

Next, consider that $K < a_1$. If we don't use surveillance, then the optimal strategy is to always intervene, and we incur an expected cost per unit time equal to $L_0(K;1)$. We set $L_0(K;1) = L_2(S_{1-}, K; 0, 1, 1, 1)$ and solve for S_{1-}:

$$S_{1-} = K - a_1 + \Delta_{10} + \Delta_{01} + \Delta_{11}. \tag{S23}$$

If $S_2 < S_{1-}$, then the benefit of appropriately intervening whenever we do not observe 00 outweighs the cost of surveillance, and surveillance using two bits is beneficial relative to always intervening. If $S_2 > S_{1-}$, then surveillance using two bits is too expensive relative to always intervening. From Equation (S23), S_{1-} is plotted versus K as the upper left boundary of the triangular region in Figure 4C.

We must also determine if surveillance using two bits is superior to surveillance using one bit. If K is between two values, which we denote K_{2+} and K_{2-}, then we should intervene if and only if we observe 1. K_{2+} is equal to the value of K for which $L_2(S_1, K_{2+}; 0, 0, 0, 1) = L_1(S_1, K_{2+}; 0, 1)$. We find

$$K_{2+} = \left[1 + \left(\frac{1}{K_+} - 1\right)\left(\frac{q_{00}p_{00} + q_{01}p_{10}}{q_{11}p_{10} + q_{10}p_{00}}\right)\right]^{-1}. \tag{S24}$$

K_{2-} is equal to the value of K for which $L_2(S_1, K_{2-}; 0, 1, 1, 1) = L_1(S_1, K_{2-}; 0, 1)$. We find

$$K_{2-} = \left[1 + \left(\frac{1}{K_-} - 1\right)\left(\frac{q_{00}p_{01} + q_{01}p_{11}}{q_{11}p_{11} + q_{10}p_{01}}\right)\right]^{-1}. \tag{S25}$$

For $K > K_{2+}$ from Equation (S24), surveillance using two bits is superior to surveillance using one bit if $S_2 - S_1 < S_{2+}$, where S_{2+} is given by $L_2(S_{2+}, K; 0, 0, 0, 1) = L_1(0, K; 0, 1)$. We have

$$S_{2+} = \Delta_{11} - \Delta_1. \tag{S26}$$

For $K < K_{2-}$ from Equation (S25), surveillance using two bits is superior to surveillance using one bit if $S_2 - S_1 < S_{2-}$, where S_{2-} is given by $L_2(S_{2-}, K; 0, 1, 1, 1) = L_1(0, K; 0, 1)$. We have

$$S_{2-} = \Delta_{10} + \Delta_{01} + \Delta_{11} - \Delta_1. \tag{S27}$$

From Equations (S26) and (S27), S_{2+} and S_{2-} are plotted versus K as the right and left boundary lines, respectively, in Figure 4D.

4 Surveillance using any number of bits of data

When considering surveillance using more than two bits of data, calculation of the optimal strategy is more complicated. For example, suppose that we are using the three most recent bits of data to inform our intervention strategy, and consider two possible bit sequences: 001 and 110. If the three most recent bits are 001, then does the most recent observation of 1 outweigh the two prior observations of 00, or vice versa? Similarly, if the three most recent bits are 110, then does the most recent observation of 0 outweigh the two prior observations of 11, or vice versa?

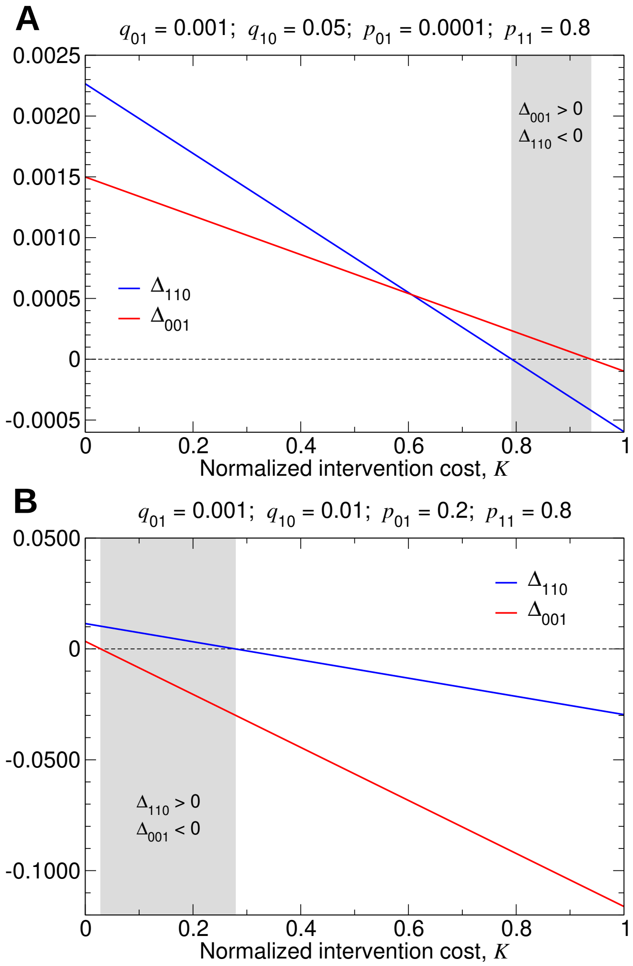

Figure S4: **Surveillance using three bits of data.** In (**A**), for intervention costs within the gray region, we should intervene if we observe 001 and not intervene if we observe 110. In (**B**), for intervention costs within the gray region, we should intervene if we observe 110 and not intervene if we observe 001. This example illustrates that optimization of surveillance and intervention using more than two bits of data can be intricate.

Figure S4 shows two possibilities for different parameter sets. First, consider Figure S4A for the case of large intervention costs (inside the gray region). A 1 in the most recent measurement outweighs 0 readings in both of the two previous measurements, and we should

intervene if we observe 001. Also, a 0 in the most recent measurement outweighs 1 readings in both of the two previous measurements, and we should not intervene if we observe 110. Thus, the analysis simplifies: Since a measurement of 001 warrants intervention, measurements of 101, 011, or 111 also warrant intervention. Furthermore, since a measurement of 110 should not induce an intervention, measurements of 010, 100, or 000 also should not induce an intervention. The conclusion is that the optimal strategy is to use only the most recent bit of data, and we should intervene if and only if this bit is 1.

By contrast, consider Figure S4B for small intervention costs (inside the gray region). Two previous 1 readings in succession outweigh a 0 in the most recent measurement, and we should intervene if we observe 110. Also, two previous 0 readings in succession outweigh a 1 in the most recent measurement, and we should not intervene if we observe 001. Using the three most recent bits of data, the optimal strategy is that we should intervene if and only if we observe 110, 101, 011, or 111. This example shows that care must be taken when determining the optimal strategy if more than two bits of data are being used.

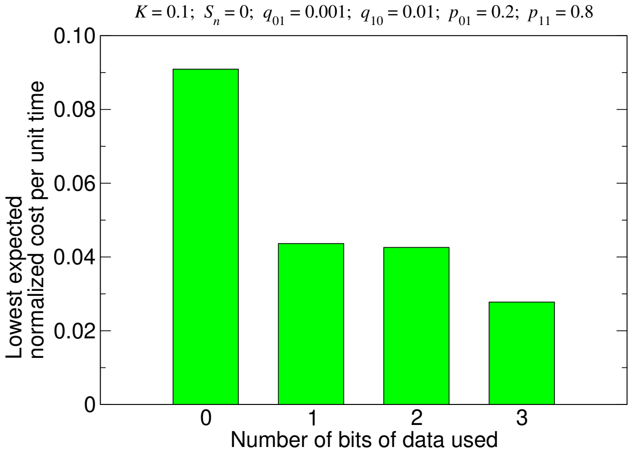

Figure S5: **Determining the optimal amount of data to use.** Consider the lowest expected normalized cost per unit time that is achievable by using zero, one, two, or three bits of data in the decision to intervene. For this example, using one bit of data yields a large improvement over no surveillance, using two bits of data yields a marginal improvement over using one bit of data, and using three bits of data yields a large improvement over using two bits of data.

A further consideration is the number of bits of data to use for deciding whether to intervene. For the same parameter values as in Figure S4B, Figure S5 shows the lowest expected normalized cost per unit time that is achievable when using zero, one, two, or three bits of data. Using zero bits of data corresponds to no surveillance and no intervention, and the resulting expected normalized cost per unit time provides a benchmark. By using just a single bit of data, and by intervening if and only if we observe 1, we achieve a $> 50\%$ reduction in the expected normalized cost per unit time. Using two bits of data and intervening if and only if we observe 11 delivers only a marginal benefit over using one bit of data. However, using three bits of data delivers a dramatic improvement in performance over using two bits of data. Determination of the optimal number of bits of data to use is therefore not always an intuitive exercise, and care must be taken in doing so.

5 Code

This section contains the code for generating the system state bits and surveillance data bits shown in Figures 2, 3, S1, S2, and S3. For Figures 2, 3, S1, S2, and S3, we set $q_{01} = 0.1$ and $q_{10} = 0.3$. For Figure 2, we set $p_{01} = 0$ and $p_{11} = 1$, while for Figures 3, S1, S2, and S3, we set $p_{01} = 0.1$ and $p_{11} = 0.9$.

The file **figure_data.c**, shown in the following subsection, contains the C code for generating the data. It uses the GNU Scientific Library (GSL) and is compiled using GCC:

```
gcc -lgsl -lgslcblas -o figure_data figure_data.c
```

Running the program produces the following data files:

- **Fig_2_state.txt**: System state bit sequence for Figure 2
- **Fig_2_surv.txt**: Surveillance bit sequence for Figure 2
- **Fig_3_S1_S2_S3_state.txt**: System state bit sequence for Figures 3, S1, S2, and S3
- **Fig_3_S1_S2_S3_surv.txt**: Surveillance bit sequence for Figures 3, S1, S2, and S3

5.1 figure_data.c

```
#include <stdio.h>
#include <gsl/gsl_rng.h>

#define N 2
#define SEED 1
#define BITS 42

int main()
{
  double q01 = 0.1, q10 = 0.3, p01[N] = {0, 0.1}, p11[N] = {1, 0.9};
  char f_state[N][25] = {"Fig_2_state.txt", "Fig_3_S1_S2_S3_state.txt"};
  char f_surv[N][24] = {"Fig_2_surv.txt", "Fig_3_S1_S2_S3_surv.txt"};

  int st, data;
  FILE *F_state, *F_surv;

  gsl_rng *mt = gsl_rng_alloc (gsl_rng_mt19937);

  for (int n=0; n<N; n++)
  {
    gsl_rng_set (mt, SEED);

    F_state = fopen (f_state[n], "w"); F_surv = fopen (f_surv[n], "w");

    st = gsl_rng_uniform(mt) < q01/(q01+q10) ? 1 : 0;
    fprintf (F_state, "%d", st);

    for (int bit=0;;)
    {
      data = gsl_rng_uniform(mt) < (st==0 ? p01[n] : p11[n]) ? 1 : 0;
      fprintf (F_surv, "%d", data);

      if (++bit==BITS) break;

      st = gsl_rng_uniform(mt) < (st==0 ? q01 : q10) ? (st+1)%2 : st;
      fprintf (F_state, "%d", st);
    }
    fprintf (F_state, "\n"); fprintf (F_surv, "\n");
    fclose (F_surv); fclose (F_state);
  }
  return 0;
}
```
